# Supplementary material for: Maternal and child gluten intake and association with type 1 diabetes: The Norwegian Mother and Child Cohort Study
Source: PLoS Med. 2020 Mar 2;17(3):e1003032. doi: 10.1371/journal.pmed.1003032 (PMC7051049; doi:10.1371/journal.pmed.1003032)
Supplement: S1 STROBE Checklist — (DOC) [file pmed.1003032.s001.doc]

**S1 STROBE Checklist**

|  | Item No | Recommendation |  | Section/Paragraph |  |
| --- | --- | --- | --- | --- | --- |
| **Title and abstract** | 1 | (*a*) Indicate the study’s design with a commonly used term in the title or the abstract |  | Title page (page 1) |  |
|  |  | (*b*) Provide in the abstract an informative and balanced summary of what was done and what was found |  | Abstract section (page 2) |  |
| Introduction | | |  |  |  |
| Background/rationale | 2 | Explain the scientific background and rationale for the investigation being reported |  | Introduction section, paragraph 1, 2 and 3 |  |
| Objectives | 3 | State specific objectives, including any prespecified hypotheses |  | Introduction section, paragraph 4 |  |
| Methods | | |  |  |  |
| Study design | 4 | Present key elements of study design early in the paper |  | Methods, “Participants and study design” section |  |
| Setting | 5 | Describe the setting, locations, and relevant dates, including periods of recruitment, exposure, follow-up, and data collection |  | Methods, “Participants and study design” and “Exposure” sections, and Fig 1 |  |
| Participants | 6 | (*a*) Give the eligibility criteria, and the sources and methods of selection of participants. Describe methods of follow-up |  | Methods, “Participants and study design”, “Exposure”, “Outcome” and “Other variables” sections, and Fig 1 |  |
|  |  | (*b*) For matched studies, give matching criteria and number of exposed and unexposed |  | N.A. |  |
| Variables | 7 | Clearly define all outcomes, exposures, predictors, potential confounders, and effect modifiers. Give diagnostic criteria, if applicable |  | Methods, “Exposure”, “Outcome” and “Other variables” sections |  |
| Data sources/ measurement | 8* | For each variable of interest, give sources of data and details of methods of assessment (measurement). Describe comparability of assessment methods if there is more than one group |  | Methods, “Participants and study design”, “Exposure”, “Outcome” and “Other variables” sections |  |
| Bias | 9 | Describe any efforts to address potential sources of bias |  | Methods, “Statistical analysis” section, paragraph 1 and 3, and Discussion, “Strengths and weaknesses” section |  |
| Study size | 10 | Explain how the study size was arrived at |  | Figure 1 |  |
| Quantitative variables | 11 | Explain how quantitative variables were handled in the analyses. If applicable, describe which groupings were chosen and why |  | Methods, “Exposure”, “Other variables” and “Statistical analysis” sections |  |
| Statistical methods | 12 | (*a*) Describe all statistical methods, including those used to control for confounding |  | Methods, “Statistical analysis” section |  |
|  |  | (*b*) Describe any methods used to examine subgroups and interactions |  | Methods, “Statistical analysis” section, paragraph 3 |  |
|  |  | (*c*) Explain how missing data were addressed |  | Methods, “Exposure”, “Outcome” and “Statistical analysis” section, Figure 1, Table 2, Supplementary Table 2, Supplementary Table 3 and Supplementary Figure 2 |  |
|  |  | (*d*) If applicable, explain how loss to follow-up was addressed |  | Figure 1 |  |
|  |  | (*e*) Describe any sensitivity analyses |  | Methods, “Statistical analysis” section, paragraph 3, and Supplementary Table3 |  |
| Results | | |  |  |  |
| Participants | 13* | (a) Report numbers of individuals at each stage of study—eg numbers potentially eligible, examined for eligibility, confirmed eligible, included in the study, completing follow-up, and analysed |  | Figure 1 and Table 2 |  |
|  |  | (b) Give reasons for non-participation at each stage |  | Figure 1 |  |
|  |  | (c) Consider use of a flow diagram |  | Figure 1 |  |
| Descriptive data | 14* | (a) Give characteristics of study participants (eg demographic, clinical, social) and information on exposures and potential confounders |  | Table 1 and Supplementary Table 1 |  |
|  |  | (b) Indicate number of participants with missing data for each variable of interest |  | Table 1 and Supplementary Table 1 |  |
|  |  | (c) Summarise follow-up time (eg, average and total amount) |  | Results section, paragraph 1 |  |
| Outcome data | 15* | Report numbers of outcome events or summary measures over time |  | Results section, paragraph 1 |  |
| Main results | 16 | (*a*) Give unadjusted estimates and, if applicable, confounder-adjusted estimates and their precision (eg, 95% confidence interval). Make clear which confounders were adjusted for and why they were included |  | Table 2 |  |
|  |  | (*b*) Report category boundaries when continuous variables were categorized |  | Table 2 |  |
|  |  | (*c*) If relevant, consider translating estimates of relative risk into absolute risk for a meaningful time period |  | Results section, paragraph 1, and Table 2 |  |
| Other analyses | 17 | Report other analyses done—eg analyses of subgroups and interactions, and sensitivity analyses |  | Results, “Additional analyses” section, Table 2 (Model 3), and Supplementary Tables 3-6 |  |
| Discussion | | |  |  |  |
| Key results | 18 | Summarise key results with reference to study objectives |  | Discussion section, paragraph 1 |  |
| Limitations | 19 | Discuss limitations of the study, taking into account sources of potential bias or imprecision. Discuss both direction and magnitude of any potential bias |  | Discussion, “Strengths and weaknesses” section |  |
| Interpretation | 20 | Give a cautious overall interpretation of results considering objectives, limitations, multiplicity of analyses, results from similar studies, and other relevant evidence |  | Discussion, “Strengths and weaknesses”, “Comparison with previous studies” and “Implications for research, clinical practice and public policy” sections |  |
| Generalisability | 21 | Discuss the generalisability (external validity) of the study results |  | Discussion, “Comparison with previous studies” sections |  |
| Other information | | |  |  |  |
| Funding | 22 | Give the source of funding and the role of the funders for the present study and, if applicable, for the original study on which the present article is based |  | Funding information will be applied from the submission form |  |

*Give information separately for exposed and unexposed groups.

**Note:** An Explanation and Elaboration article discusses each checklist item and gives methodological background and published examples of transparent reporting. The STROBE checklist is best used in conjunction with this article (freely available on the Web sites of PLoS Medicine at http://www.plosmedicine.org/, Annals of Internal Medicine at http://www.annals.org/, and Epidemiology at http://www.epidem.com/). Information on the STROBE Initiative is available at http://www.strobe-statement.org.
