# Supplementary material for: Maternal and child gluten intake and association with type 1 diabetes: The Norwegian Mother and Child Cohort Study
Source: PLoS Med. 2020 Mar 2;17(3):e1003032. doi: 10.1371/journal.pmed.1003032 (PMC7051049; doi:10.1371/journal.pmed.1003032)
Supplement: S1 Table — (DOCX) [file pmed.1003032.s002.docx]

**S1 Table. Characteristics of the study participants included in the analysis of the child’s gluten intake at age 18 months and risk of type 1 diabetes**

| **Gluten intake by percentile median(range) in g/day** | **All participants**  **8.8 (0-34.9)** | **<10%**  **3.9 (0-4.8)** | **10-20%**  **5.3 (4.8-5.8)** | | **20-50%**  **7.0 (5.8-8.2)** | **50-80%**  **9.6 (8.2-11.4)** | **80-90%**  **12.3 (11.4-13.5)** | **>90%**  **16.3 (13.5-34.9)** |
| --- | --- | --- | --- | --- | --- | --- | --- | --- |
| **Maternal characteristics** | *n = 66 725* | *n = 6524* | | *n = 6636* | *n = 20 152* | *n = 20 078* | *n = 6693* | *n = 6642* |
| **Age in years, mean (SD)** | 30.4 (4.4) | 29.8 (4.8) | | 30.0 (4.6) | 30.3 (4.4) | 30.6 (4.3) | 30.7 (4.3) | 30.8 (4.4) |
| <25 | 6145 (9.2) | 933 (14.3) | | 775 (11.7) | 1901 (9.4) | 1600 (8.0) | 453 (6.8) | 483 (7.3) |
| 25-34 | 48 622 (72.9) | 4503 (69.0) | | 4768 (71.9) | 14 756 (73.2) | 14 811 (73.8) | 4963 (74.2) | 4821 (72.6) |
| ≥35 | 11 958 (17.9) | 1088 (16.7) | | 1093 (16.5) | 3495 (17.3) | 3667 (18.3) | 1277 (19.1) | 1338 (20.1) |
| **Pre-pregnancy BMI, mean (SD)** | 24.0 (4.2) | 24.3 (4.5) | | 24.2 (4.3) | 24.1 (4.2) | 23.9 (4.1) | 23.8 (4.0) | 23.7 (4.0) |
| <20 | 8090 (12.1) | 766 (11.7) | | 744 (11.2) | 2356 (11.7) | 2454 (12.2) | 868 (13.0) | 902 (13.6) |
| 20-25 | 36 979 (55.4) | 3424 (52.5) | | 3597 (54.2) | 11 124 (55.2) | 11 351 (56.5) | 3738 (55.8) | 3745 (56.4) |
| 25-29.9 | 14 196 (21.3) | 1400 (21.5) | | 1479 (22.3) | 4347 (21.6) | 4217 (21.0) | 1400 (20.9) | 1353 (20.4) |
| ≥30 | 5941 (8.9) | 717 (11.0) | | 655 (9.9) | 1877 (9.3) | 1636 (8.1) | 542 (8.1) | 514 (7.7) |
| *Missing data* | 1519 (2.3) | 217 (3.3) | | 161 (2.4) | 448 (2.2) | 420 (2.1) | 145 (2.2) | 128 (1.9) |
| **Prematurity** | 3903 (5.8) | 364 (5.6) | | 352 (5.3) | 1112 (5.5) | 1218 (6.1) | 434 (6.5) | 423 (6.4) |
| *Missing data* | 10 (0.0) | 1 (0.0) | | 1 (0.0) | 2 (0.0) | 6 (0.0) | - | - |
| **Parity** |  |  | |  |  |  |  |  |
| 0 | 31 370 (47.0) | 2979 (45.7) | | 3045 (45.9) | 9379 (46.5) | 9508 (47.4) | 3263 (48.8) | 3196 (48.1) |
| 1 | 23 054 (34.6) | 2259 (34.6) | | 2366 (35.7) | 6985 (34.7) | 6917 (34.5) | 2230 (33.3) | 2297 (34.6) |
| ≥2 | 12 301 (18.4) | 1286 (19.7) | | 1225 (18.5) | 3788 (18.8) | 3653 (18.2) | 1200 (17.9) | 1149 (17.3) |
| **Smoking during pregnancy** |  |  | |  |  |  |  |  |
| No | 61 213 (91.7) | 5748 (88.1) | | 6005 (90.5) | 18 466 (91.6) | 18 633 (92.8) | 6243 (93.3) | 6118 (92.1) |
| Occasionally | 1077 (1.6) | 120 (1.8) | | 107 (1.6) | 334 (1.7) | 325 (1.6) | 88 (1.3) | 103 (1.6) |
| Yes | 4105 (6.2) | 621 (9.5) | | 496 (7.5) | 1251 (6.2) | 1027 (5.1) | 327 (4.9) | 383 (5.8) |
| *Missing data* | 330 (0.5) | 35 (0.5) | | 28 (0.4) | 101 (0.5) | 93 (0.5) | 35 (0.5) | 38 (0.6) |
| **Maternal Education** |  |  | |  |  |  |  |  |
| <12 years | 22 273 (33.4) | 2955 (45.3) | | 2550 (38.4) | 6758 (33.5) | 6015 (30.0) | 1922 (28.7) | 2073 (31.2) |
| 12-15 years | 28 133 (42.2) | 2346 (36.0) | | 2642 (39.8) | 8603 (42.7) | 8863 (44.1) | 2948 (44.0) | 2731 (41.1) |
| ≥16 years | 16 060 (24.1) | 1186 (18.2) | | 1416 (21.3) | 4723 (23.4) | 5130 (25.6) | 1795 (26.8) | 1810 (27.3) |
| *Missing data* | 259 (0.4) | 37 (0.6) | | 28 (0.4) | 68 (0.3) | 70 (0.3) | 28 (0.4) | 28 (0.4) |
| **Breastfeeding duration, mean (SD)** | 9.9 (4.7) | 9.2 (4.7) | | 9.5 (4.5) | 9.8 (4.5) | 10.1 (4.4) | 10.2 (4.4) | 10.2 (4.5) |
| <6.0 months | 13 329 (20.0) | 1702 (26.1) | | 1480 (22.3) | 4069 (20.2) | 3666 (18.3) | 1185 (17.7) | 1227 (18.5) |
| 6.0-11.9 months | 27 715 (41.5) | 2634 (40.4) | | 2873 (43.3) | 8533 (42.3) | 8287 (41.3) | 2776 (41.5) | 2612 (39.3) |
| ≥12.0 months | 25 681 (38.5) | 2188 (33.5) | | 2283 (34.4) | 7550 (37.5) | 8125 (40.5) | 2732 (40.8) | 2803 (42.2) |
| **Fibre intake(g), mean (SD)** | 30.8 (11.1) | 28.8 (11.3) | | 29.2 (10.6) | 29.8 (10.4) | 31.3 (10.9) | 32.3 (11.1) | 34.0 (12.6) |
| <20th centile | 12 267 (18.4) | 1741 (26.7) | | 1560 (23.5) | 4069 (20.2) | 3283 (16.4) | 863 (12.9) | 751 (11.3) |
| 20-40th centile | 13 192 (19.8) | 1375 (21.1) | | 1401 (21.1) | 4300 (21.3) | 3807 (19.0) | 1256 (18.8) | 1053 (15.9) |
| 40-60th centile | 13 478 (20.2) | 1183 (18.1) | | 1263 (19.0) | 4106 (20.4) | 4160 (20.7) | 1411 (21.1) | 1355 (20.4) |
| 60-80th centile | 13 404 (20.1) | 1052 (16.1) | | 1226 (18.5) | 3871 (19.2) | 4283 (21.3) | 1484 (22.2) | 1488 (22.4) |
| 80-100th centile | 13 150 (19.7) | 1051 (16.1) | | 1070 (16.1) | 3441 (17.1) | 4158 (20.7) | 1554 (23.2) | 1876 (28.2) |
| *Missing data* | 1234 (1.8) | 122 (1.9) | | 116 (1.7) | 365 (1.8) | 387 (1.9) | 125 (1.9) | 119 (1.8) |
| **Energy intake (MJ), mean (SD)** | 9.7 (2.9) | 9.2 (3.1) | | 9.3 (2.8) | 9.4 (2.8) | 9.8 (2.9) | 10.0 (2.9) | 10.4 (3.2) |
| <20th centile | 12 425 (18.6) | 1756 (26.9) | | 1538 (23.2) | 4058 (20.1) | 3298 (16.4) | 956 (14.3) | 819 (12.3) |
| 20-40th centile | 13 257 (19.9) | 1334 (20.4) | | 1389 (20.9) | 4277 (21.2) | 3933 (19.6) | 1221 (18.2) | 1103 (16.6) |
| 40-60th centile | 13 512 (20.3) | 1206 (18.5) | | 1317 (19.8) | 4099 (20.3) | 4173 (20.8) | 1390 (20.8) | 1327 (20.0) |
| 60-80th centile | 13 428 (20.1) | 1072 (16.4) | | 1179 (17.8) | 3963 (19.7) | 4245 (21.1) | 1481 (22.1) | 1488 (22.4) |
| 80-100th centile | 12 869 (19.3) | 1034 (15.8) | | 1097 (16.5) | 3390 (16.8) | 4042 (20.1) | 1520 (22.7) | 1786 (26.9) |
| *Missing data* | 1234 (1.8) | 122 (1.9) | | 116 (1.7) | 365 (1.8) | 387 (1.9) | 125 (1.9) | 119 (1.8) |
| **Offspring characteristics** |  |  | |  |  |  |  |  |
| **Caesarean section** | 9668 (14.5) | 979 (15.0) | | 946 (14.3) | 2849 (14.1) | 2831 (14.1) | 990 (14.8) | 1073 (16.2) |
| **Type 1 Diabetes** | 271 (0.4) | 19 (0.3) | | 25 (0.4) | 79 (0.4) | 80 (0.4) | 31 (0.5) | 37 (0.6) |
| **Female** | 32 640 (48.9) | 3284 (50.3) | | 3332 (50.2) | 9989 (49.6) | 9694 (48.3) | 3215 (48.0) | 3126 (47.1) |
| **Birthweight (g), mean (SD)** | 3570 (577) | 3568 (579) | | 3583 (562) | 3576 (572) | 3567 (578) | 3567 (582) | 3557 (592) |
| <2500 | 2552 (3.8) | 228 (3.5) | | 223 (3.4) | 718 (3.6) | 810 (4.0) | 285 (4.3) | 288 (4.3) |
| 2500-3499 | 25 595 (38.4) | 2574 (39.5) | | 2511 (37.8) | 7777 (38.6) | 7640 (38.1) | 2539 (37.9) | 2554 (38.5) |
| 3500-4499 | 35 745 (53.6) | 3428 (52.5) | | 3619 (54.5) | 10 785 (53.5) | 10 810 (53.8) | 3570 (53.3) | 3533 (53.2) |
| >4500 | 2832 (4.2) | 294 (4.5) | | 283 (4.3) | 872 (4.3) | 817 (4.1) | 299 (4.5) | 267 (4.0) |
| *Missing data* | 1 (0.0) | - | | - | - | 1 (0.0) | - | - |
| **Age at gluten introduction** |  |  | |  |  |  |  |  |
| <4.0 months | 438 (0.7) | 72 (1.1) | | 59 (0.9) | 137 (0.7) | 93 (0.5) | 32 (0.5) | 45 (0.7) |
| 4.0-5.9 months | 15 015 (22.5) | 1560 (23.9) | | 1586 (23.9) | 4644 (23.0) | 4379 (21.8) | 1398 (20.9) | 1448 (21.8) |
| ≥6.0 months | 51 272 (76.8) | 4892 (75.0) | | 4991 (75.2) | 15 371 (76.3) | 15 606 (77.7) | 5263 (78.6) | 5149 (77.5) |
| **Coeliac disease diagnosis** | 666 (1.0) | 55 (0.8) | | 56 (0.8) | 173 (0.9) | 232 (1.2) | 72 (1.1) | 78 (1.2) |
| *Missing data* | 122 (0.2) | 18 (0.3) | | 11 (0.2) | 33 (0.2) | 37 (0.2) | 10 (0.1) | 13 (0.2) |
| **Weight Gain (kg) 0-12 months, mean (SD)** | 6.4 (1.1) | 6.4 (1.1) | | 6.4 (1.1) | 6.4 (1.1) | 6.4 (1.0) | 6.3 (1.0) | 6.3 (1.0) |
| *Missing data* | 7792 (11.7) | 919 (14.1) | | 867 (13.1) | 2301 (11.2) | 2241 (11.4) | 764 (11.4) | 700 (10.5) |
