# Supplementary material for: Maternal and child gluten intake and association with type 1 diabetes: The Norwegian Mother and Child Cohort Study
Source: PLoS Med. 2020 Mar 2;17(3):e1003032. doi: 10.1371/journal.pmed.1003032 (PMC7051049; doi:10.1371/journal.pmed.1003032)
Supplement: S2 Table — (DOCX) [file pmed.1003032.s003.docx]

**S2 Table. Characteristics of excluded participants due to missing exposure data**

| **Excluded in maternal analysis** | **Excluded participants, pregnancy intake** | **Excluded participants, childhood intake** |
| --- | --- | --- |
| **Maternal characteristics** | *n =14 522* | *n = 34 103* |
| **Age in years, mean (SD)** | 29.9 (4.7) | 29.8 (4.8) |
| <25 | 1881 (13.0) | 4901 (14.4) |
| 25-34 | 10 284 (70.8) | 23 474 (68.8) |
| ≥35 | 2357 (16.2) | 5728 (16.8) |
| **Pre-pregnancy BMI, mean (SD)** | 23.9 (4.2) | 24.1 (4.4) |
| <20 | 1927 (13.3) | 4359 (12.8) |
| 20-25 | 7845 (54.0) | 17 898 (52.5) |
| 25-29.9 | 2942 (20.3) | 7221 (21.2) |
| ≥30 | 1281 (8.8) | 3395 (10.0) |
| *Missing data* | 527 (3.6) | 1230 (3.6) |
| **Prematurity** | 824 (5.7) | 2312 (6.8) |
| *Missing data* | 39 (0.3) | 90 (0.3) |
| **Parity** |  |  |
| 0 | 5724 (39.4) | 13 902 (40.8) |
| 1 | 5448 (37.5) | 12 841 (37.7) |
| ≥2 | 3350 (23.1) | 7360 (21.6) |
| **Smoking during pregnancy** |  |  |
| No | 11 327 (78.0) | 28 446 (83.4) |
| Occasionally | 403 (2.8) | 791 (2.3) |
| Yes | 1677 (11.5) | 3616 (10.6) |
| *Missing data* | 1115 (7.7) | 1250 (3.7) |
| **Maternal Education** |  |  |
| <12 years | 6533 (45.0) | 15 376 (45.1) |
| 12-15 years | 5297 (36.5) | 12 146 (35.6) |
| ≥16 years | 2605 (17.9) | 6334 (18.6) |
| *Missing data* | 87 (0.6) | 247 (0.7) |
| **Breastfeeding duration, mean(SD)** | 10.0 (4.4) | 10.0 (4.4) |
| <6.0 months | 1574 (10.8) | 946 (2.8) |
| 6.0-11.9 months | 3646 (25.1) | 2157 (6.3) |
| ≥12 months | 3322 (22.9) | 1945 (5.7) |
| *Missing data* | 5980 (41.2) | 29 055 (85.2) |
| **Fibre intake(g), mean(SD)** | 25.0 (8.3) | 30.4 (13.2) |
| <20th centile | 3277 (22.6) | 6786 (19.9) |
| 20-40th centile | 2389 (16.5) | 5916 (17.3) |
| 40-60th centile | 1645 (11.3) | 5585 (16.4) |
| 60-80th centile | 1104 (7.6) | 5631 (16.5) |
| 80-100th centile | 530 (3.6) | 5841 (17.1) |
| *Missing data* | 5577 (38.4) | 4344 (12.7) |
| **Energy intake (MJ), mean(SD)** | 8.3 (2.3) | 9.7 (3.7) |
| <20th centile | 3243 (22.3) | 6562 (19.2) |
| 20-40th centile | 2220 (15.3) | 5849 (17.2) |
| 40-60th centile | 1634 (11.3) | 5542 (16.3) |
| 60-80th centile | 1196 (8.2) | 5651 (16.6) |
| 80-100th centile | 652 (4.5) | 6155 (18.0) |
| *Missing data* | 5577 (38.4) | 4344 (12.7) |

Continued – S2 Table. Characteristics of excluded participants due to missing exposure data

| **Offspring characteristics** |  |  |
| --- | --- | --- |
| **Caesarean section** | 2032 (14.0) | 5365 (15.7) |
| **Type 1 Diabetes** | 66 (0.5) | 141 (0.4) |
| **Sex** |  |  |
| Male | 7050 (48.5) | 16 589 (48.6) |
| **Birthweight (g), mean (SD)** | 3598 (598) | 3558 (612) |
| <2500 | 591 (4.1) | 1624 (4.8) |
| 2500-3499 | 5208 (35.9) | 12 798 (37.5) |
| 3500-4499 | 7977 (54.9) | 18 164 (53.3) |
| ≥4500 | 736 (5.1) | 1502 (4.4) |
| *Missing data* | 10 (0.1) | 15 (0.0) |
| **Age at gluten introduction** |  |  |
| <4.0 months | 184 (1.3) | 261 (0.8) |
| 4.0-5.9 months | 4314 (29.7) | 5902 (17.3) |
| ≥6.0 months | 6124 (42.2) | 14 199 (41.6) |
| *Missing data* | 3900 (26.9) | 13 741 (40.3) |
| **Coeliac disease diagnosis** | 114 (0.8) | 276 (0.8) |
| *Missing data* | 24 (0.2) | 55 (0.2) |
| **Weight Gain (kg) 0-12 months, mean (SD)** | 6.3 (1.1) | 6.3 (1.0) |
| *Missing data* | 6825 | 27 862 |
