# Supplementary material for: Maternal and child gluten intake and association with type 1 diabetes: The Norwegian Mother and Child Cohort Study
Source: PLoS Med. 2020 Mar 2;17(3):e1003032. doi: 10.1371/journal.pmed.1003032 (PMC7051049; doi:10.1371/journal.pmed.1003032)
Supplement: S3 Table — Complete case analyses*. (DOCX) [file pmed.1003032.s004.docx]

**S3 Table. Association between maternal gluten intake during pregnancy (n = 54,332) or child’s intake at 18 months (n = 56,257) and the risk of type 1 diabetes in the child. Complete case analyses.***

| Gluten intake | Cases | Incidence rate (per 100 000) | Hazard ratio (95% CI) of type 1 diabetes | | | | | |
| --- | --- | --- | --- | --- | --- | --- | --- | --- |
|  |  |  | **Unadjusted** | **p-value** | **Adjusted model 1**^†^ | **p-value** | **Adjusted model 2**^‡^ | **p-value** |
|  |  |  |  |  |  |  | **(primary model)** |  |
| Maternal/  Pregnancy |  |  |  |  |  |  |  |  |
| Continuous,  per 10 g/day increase | 219 | 32.7 | 0.91 (0.70 – 1.18) | 0.47 | 0.98 (0.71 – 1.36) | 0.91 | 1.01 (0.69 – 1.48) | 0.95 |
| By category |  |  |  |  |  |  |  |  |
| <7.6 g/day | 15 | 25.1 | Ref. |  | Ref. |  | Ref. |  |
| 7.6-9.5 g/day | 28 | 42.3 | 1.68 (0.90 – 3.14) | 0.11 | 1.73 (0.92 – 3.25) | 0.09 | 1.59 (0.84 – 3.03) | 0.15 |
| 9.5-13.0 g/day | 74 | 35.8 | 1.42 (0.81 – 2.47) | 0.22 | 1.52 (0.86 – 2.68) | 0.15 | 1.50 (0.84 – 2.69) | 0.17 |
| 13.0-17.3 g/day | 59 | 28.7 | 1.14 (0.64 – 2.01) | 0.65 | 1.28 (0.69 – 2.38) | 0.43 | 1.26 (0.65 – 2.44) | 0.50 |
| 17.3-20.1 g/day | 22 | 32.7 | 1.29 (0.67 – 2.50) | 0.44 | 1.50 (0.74 – 3.07) | 0.26 | 1.38 (0.63 – 2.99) | 0.42 |
| >20.1 g/day | 21 | 33.1 | 1.31 (0.67 – 2.54) | 0.43 | 1.55 (0.72 – 3.31) | 0.26 | 1.68 (0.74 – 3.85) | 0.22 |
| Child/  18 Months of life |  |  |  |  |  |  |  |  |
| Continuous,  per 10 g/day increase | 218 | 35.4 | 1.33 (0.94 – 1.88) | 0.11 | 1.35 (0.96 – 1.90) | 0.08 | 1.38 (0.97 – 1.96) | 0.07 |
| By category |  |  |  |  |  |  |  |  |
| <4.8 g/day | 15 | 28.8 | Ref. |  | Ref. |  | Ref. |  |
| 4.8-5.8 g/day | 26 | 44.9 | 1.22 (0.63 – 2.35) | 0.55 | 1.23 (0.63 – 2.37) | 0.54 | 1.21 (0.63 – 2.33) | 0.57 |
| 5.8-8.2 g/day | 73 | 40.3 | 1.30 (0.75 – 2.24) | 0.35 | 1.31 (0.76 – 2.28) | 0.33 | 1.25 (0.72 – 2.17) | 0.42 |
| 8.2-11.4 g/day | 56 | 31.2 | 1.23 (0.71 – 2.13) | 0.47 | 1.26 (0.72 – 2.18) | 0.42 | 1.18 (0.68 – 2.06) | 0.55 |
| 11.4-13.5 g/day | 19 | 32.3 | 1.68 (0.94 – 3.11) | 0.09 | 1.73 (0.93 – 3.20) | 0.08 | 1.67 (0.90 – 3.11) | 0.10 |
| >13.5 g/day | 21 | 37.9 | 1.54 (0.83 – 2.87) | 0.18 | 1.59 (0.85 – 2.97) | 0.15 | 1.55 (0.83 – 2.91) | 0.17 |

* Analyses including only individuals with no missing data on any of the covariates in model 2 (to compare with main table 2 where missing covariate data were imputed).

† Model 1: Adjusted for maternal age, pre-pregnant maternal body mass index, parity, smoking during pregnancy, education, caesarean section, breastfeeding, sex and energy intake.

‡ Model 2 (primary model): As model 1 with additional adjustment for birthweight, age at gluten introduction, prematurity, fibre intake, weight gain 0-12 months and child’s or mothers gluten intake (mutually adjusted exposures).
