# Supplementary material for: Maternal and child gluten intake and association with type 1 diabetes: The Norwegian Mother and Child Cohort Study
Source: PLoS Med. 2020 Mar 2;17(3):e1003032. doi: 10.1371/journal.pmed.1003032 (PMC7051049; doi:10.1371/journal.pmed.1003032)
Supplement: S4 Table — (DOCX) [file pmed.1003032.s005.docx]

**S4 Table. Association between maternal intake of fibre during pregnancy and the risk of type 1 diabetes in the child**

| **Maternal fibre intake during pregnancy** | **Hazard ratio (95% CI) of type 1 diabetes** | | | |
| --- | --- | --- | --- | --- |
|  | **Unadjusted** | **p-value** | **Adjusted*** | **p-value** |
| Continuous, 1 g/day increase | 1.00 (0.99 - 1.01) | 0.95 | 1.00 (0.99 - 1.01) | 0.43 |
| By category |  |  |  |  |
| 0-20% | Ref. |  | Ref. |  |
| 20-40% | 1.13 (0.81 - 1.56) | 0.47 | 1.09 (0.78 - 1.52) | 0.61 |
| 40-60% | 1.04 (0.75 - 1.46) | 0.80 | 0.98 (0.69 - 1.39) | 0.91 |
| 60-80% | 0.96 (0.68 - 1.36) | 0.82 | 0.88 (0.60 - 1.28) | 0.50 |
| 80-100% | 1.01 (0.71 - 1.42) | 0.97 | 0.86 (0.56 - 1.33) | 0.50 |

* Adjusted for maternal age, pre-pregnant maternal body mass index, parity, smoking during pregnancy, education, caesarean section, breastfeeding, sex, energy intake, birthweight, age at gluten introduction, prematurity, fibre intake, weight gain 0-12 months.
