# Supplementary material for: Maternal and child gluten intake and association with type 1 diabetes: The Norwegian Mother and Child Cohort Study
Source: PLoS Med. 2020 Mar 2;17(3):e1003032. doi: 10.1371/journal.pmed.1003032 (PMC7051049; doi:10.1371/journal.pmed.1003032)
Supplement: S5 Table — (DOCX) [file pmed.1003032.s006.docx]

**S5 Table. Association between maternal intake of gluten from refined grains during pregnancy and the risk of type 1 diabetes in the child.***

| **Maternal gluten intake from refined grains** | **Hazard ratio (95% CI) of type 1 diabetes** | **p-value** |
| --- | --- | --- |
| <7.6 g/day | Ref. |  |
| 7.6-9.5 g/day | 0.83 (0.48 - 1.43) | 0.50 |
| 9.5-13.0 g/day | 0.55 (0.28 - 1.08) | 0.08 |
| 13.0-17.3 g/day | 0.84 (0.56 - 1.26) | 0.40 |
| 17.3-20.1 g/day | 0.76 (0.43 - 1.36) | 0.36 |
| >20.1 g/day | 0.82 (0.47 - 1.42) | 0.48 |

* Adjusted for maternal age, pre-pregnant maternal body mass index, parity, smoking during pregnancy, education, caesarean section, breastfeeding, sex, energy intake, birthweight, age at gluten introduction, prematurity, fibre intake, weight gain 0-12 months.
