# Supplementary material for: Maternal and child gluten intake and association with type 1 diabetes: The Norwegian Mother and Child Cohort Study
Source: PLoS Med. 2020 Mar 2;17(3):e1003032. doi: 10.1371/journal.pmed.1003032 (PMC7051049; doi:10.1371/journal.pmed.1003032)
Supplement: S6 Table — (DOCX) [file pmed.1003032.s007.docx]

**S6 Table. Analyses of model 2 (primary model) including children who have a mother with type 1 diabetes or coeliac disease (n = 1,033).***

| **Category** | **aHR, 95% CI†** | **p** |
| --- | --- | --- |
| Childhood intake |  |  |
| Per 10 grams | 1.48 (1.10 - 2.00) | 0.01 |
| <4.8 g/day | Ref. |  |
| 4.8-5.8 g/day | 1.29 (0.71 - 2.34) | 0.40 |
| 5.8-8.2 g/day | 1.32 (0.80 - 2.18) | 0.28 |
| 8.2-11.4 g/day | 1.26 (0.76 - 2.09) | 0.37 |
| 11.4-13.5 g/day | 1.62 (0.92 - 2.88) | 0.10 |
| >13.5 g/day | 1.85 (1.06 - 3.22) | 0.03 |
| Maternal intake |  |  |
| Per 10 grams | 0.98 (0.71 - 1.36) | 0.92 |
| <7.6 g/day | Ref. |  |
| 7.6-9.5 g/day | 1.72 (0.97 - 3.06) | 0.06 |
| 9.5-13.0 g/day | 1.36 (0.80 - 2.32) | 0.25 |
| 13.0-17.3 g/day | 1.17 (0.65 - 2.11) | 0.59 |
| 17.3-20.1 g/day | 1.45 (0.74 - 2.81) | 0.28 |
| >20.1 g/day | 1.46 (0.71 - 3.02) | 0.31 |

* In total n=1,682, were 649 lacked exposure data. Resulting in n=1,033 additional participants of which n=15 had type 1 diabetes.

† Adjusted for maternal age, pre-pregnant maternal body mass index, parity, smoking during pregnancy, education, caesarean section, breastfeeding, sex, energy intake, birthweight, age at gluten introduction, prematurity, fibre intake, weight gain 0-12 months and child’s or mothers gluten intake (mutually adjusted exposures).
