# Supplementary material for: Maternal and child gluten intake and association with type 1 diabetes: The Norwegian Mother and Child Cohort Study
Source: PLoS Med. 2020 Mar 2;17(3):e1003032. doi: 10.1371/journal.pmed.1003032 (PMC7051049; doi:10.1371/journal.pmed.1003032)

**S1 Fig. Excerpts of questionnaires at week 22 of pregnancy and child’s age 18 months. Full versions of the questionnaires can be found at** [**https://www.fhi.no/en/studies/moba/for-forskere-artikler/questionnaires-from-moba/**](https://www.fhi.no/en/studies/moba/for-forskere-artikler/questionnaires-from-moba/)


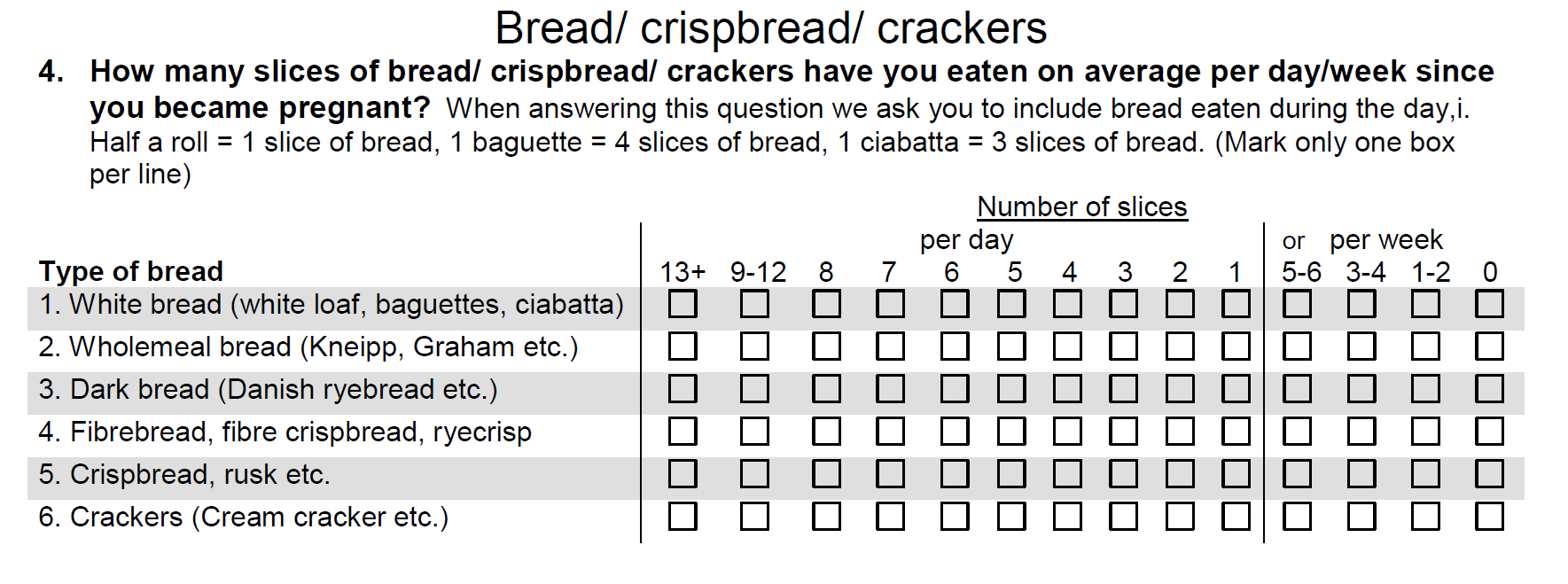


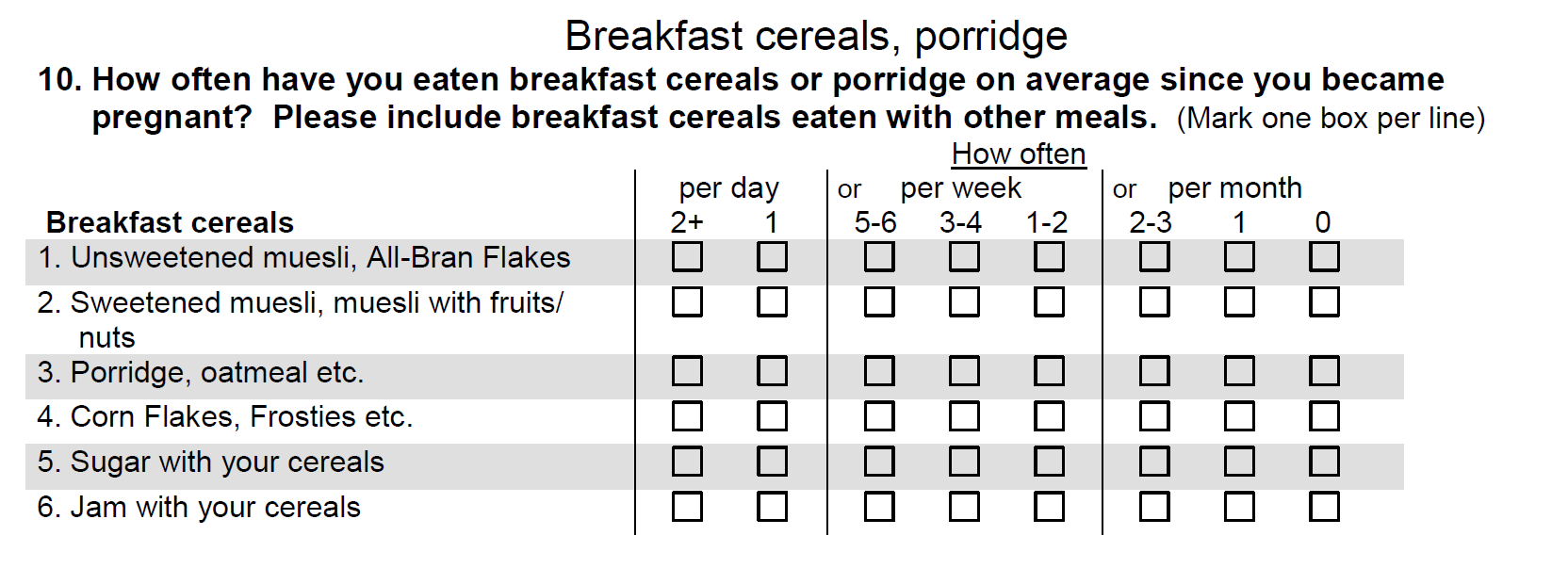


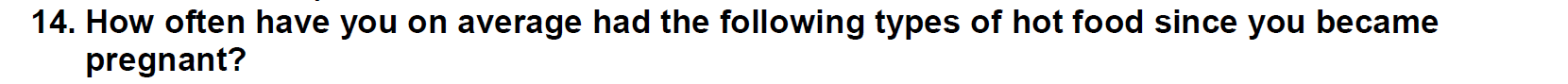


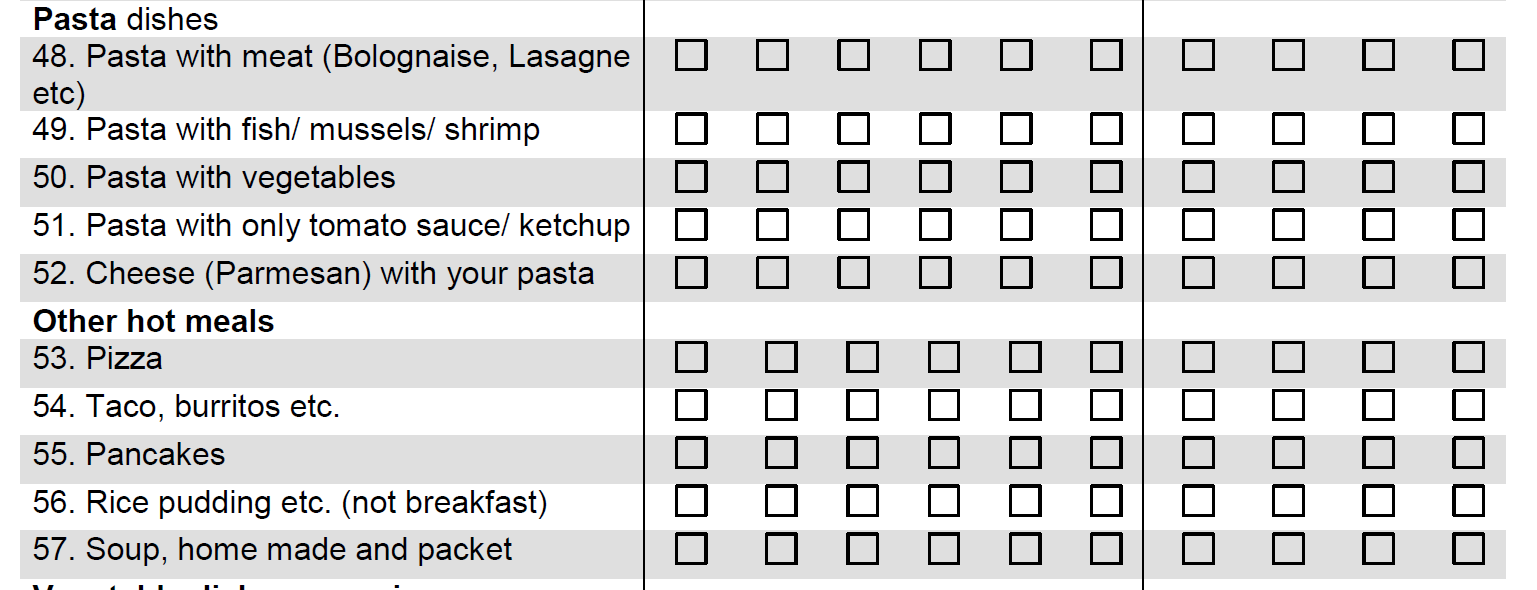


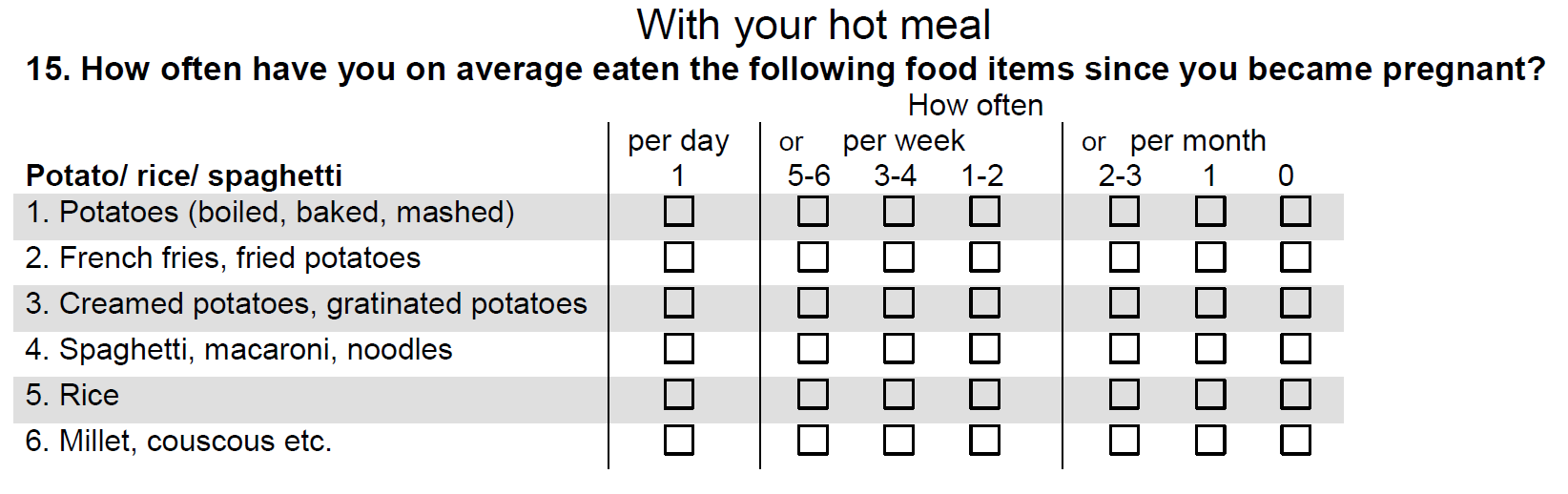


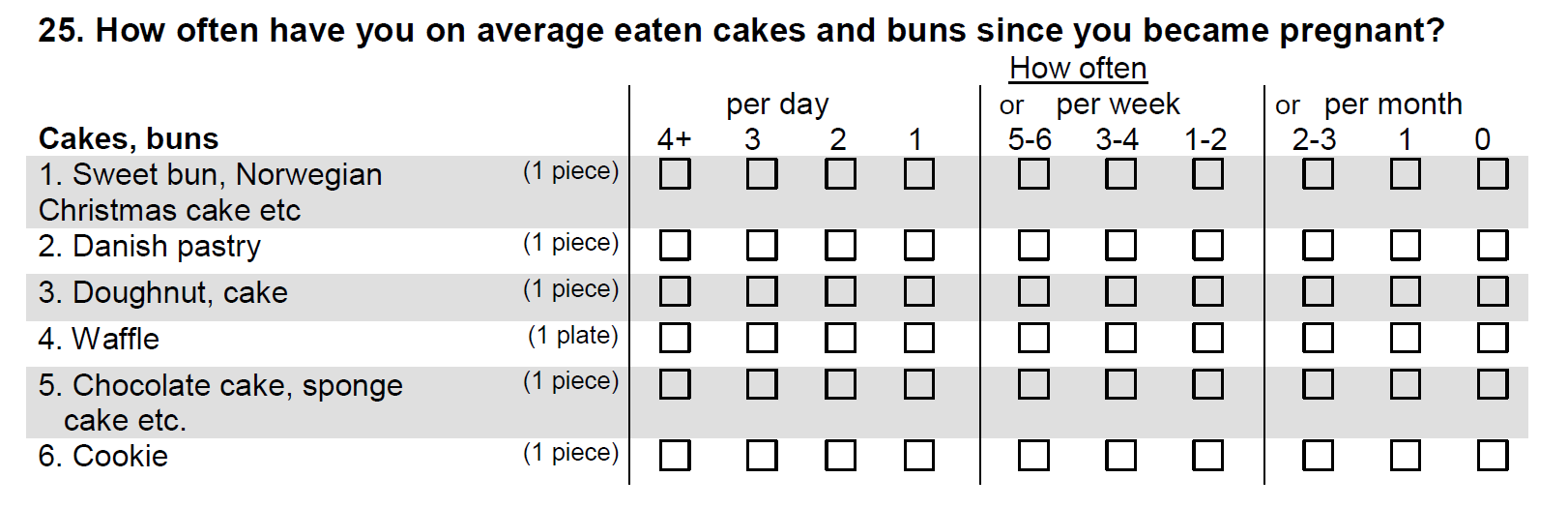


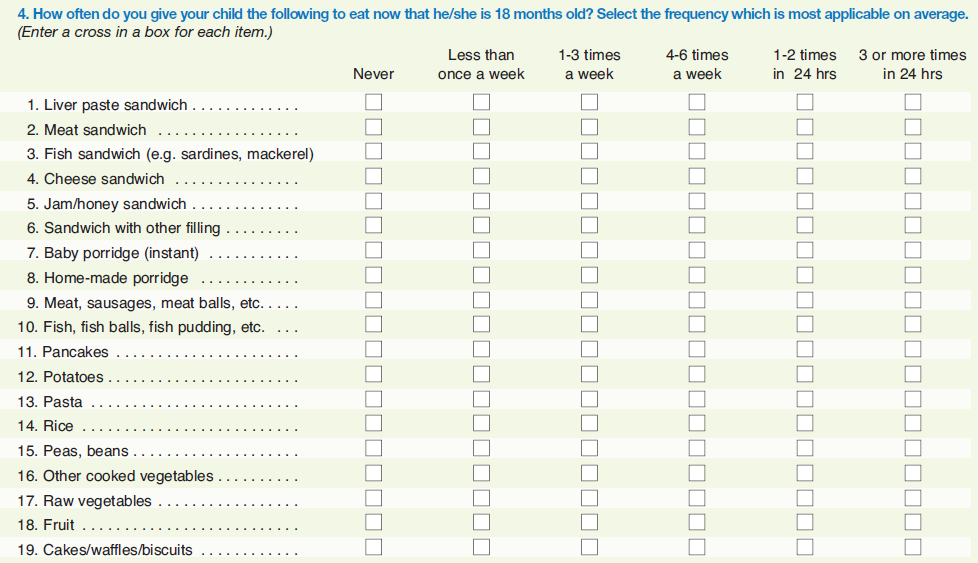

Supplement: S1 Fig — (DOCX) [file pmed.1003032.s008.docx]
