# Supplementary material for: Maternal and child gluten intake and association with type 1 diabetes: The Norwegian Mother and Child Cohort Study
Source: PLoS Med. 2020 Mar 2;17(3):e1003032. doi: 10.1371/journal.pmed.1003032 (PMC7051049; doi:10.1371/journal.pmed.1003032)
Supplement: S2 Fig — (DOCX) [file pmed.1003032.s009.docx]

**S2 Fig. Cumulative incidence of type 1 diabetes in participants included in the analyses and in those excluded due to missing gluten data**


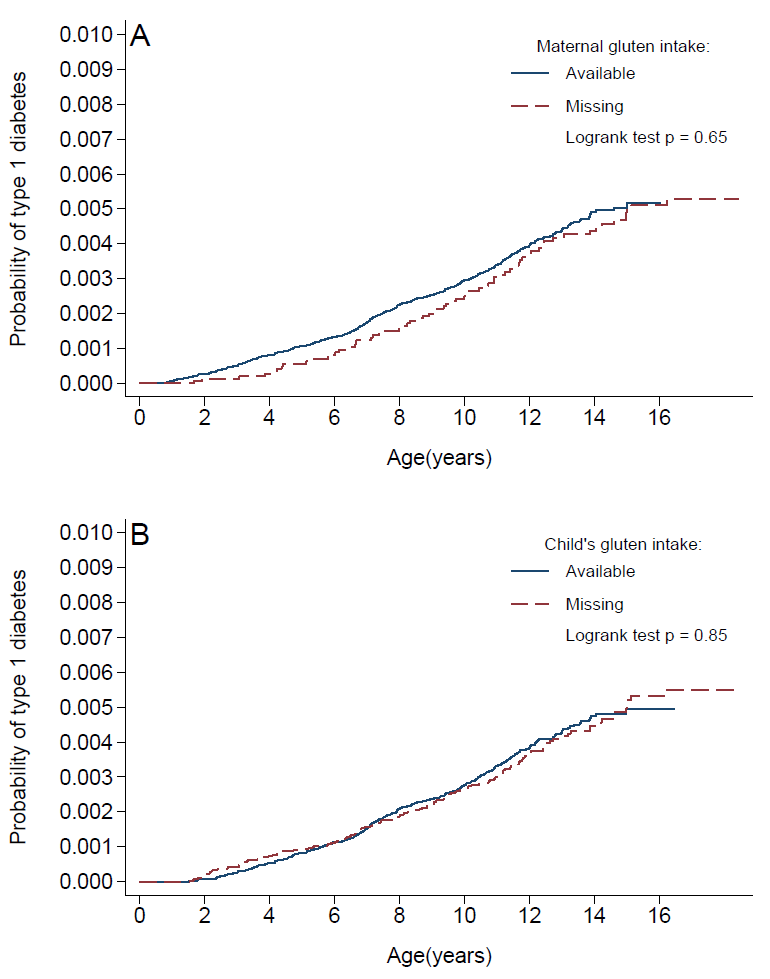


Panel A shows the survival functions of those with available and missing gluten intake data during pregnancy. Panel B shows the survival functions of those with available and missing gluten intake data during early childhood. The logrank test for equality of survivor functions show that there is no significant difference between the survivor functions in those included and excluded due to missing gluten data.
