# Supplementary material for: Maternal and child gluten intake and association with type 1 diabetes: The Norwegian Mother and Child Cohort Study
Source: PLoS Med. 2020 Mar 2;17(3):e1003032. doi: 10.1371/journal.pmed.1003032 (PMC7051049; doi:10.1371/journal.pmed.1003032)

**S3 Fig. Distribution of maternal gluten intake during pregnancy and child’s gluten intake at age 18 months.**


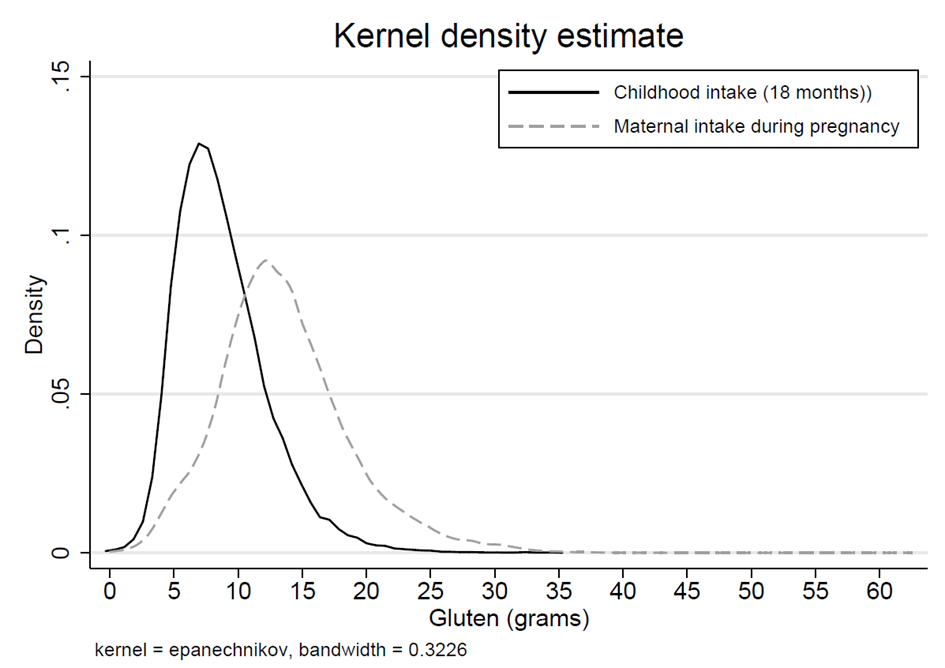

Supplement: S3 Fig — (DOCX) [file pmed.1003032.s010.docx]
