# Supplementary material for: Maternal and child gluten intake and association with type 1 diabetes: The Norwegian Mother and Child Cohort Study
Source: PLoS Med. 2020 Mar 2;17(3):e1003032. doi: 10.1371/journal.pmed.1003032 (PMC7051049; doi:10.1371/journal.pmed.1003032)

# S5 Fig. Association between maternal gluten intake during pregnancy and child’s gluten intake at age 18 months and risk of type 1 diabetes, stratified by child’s sex and birth weight.

**
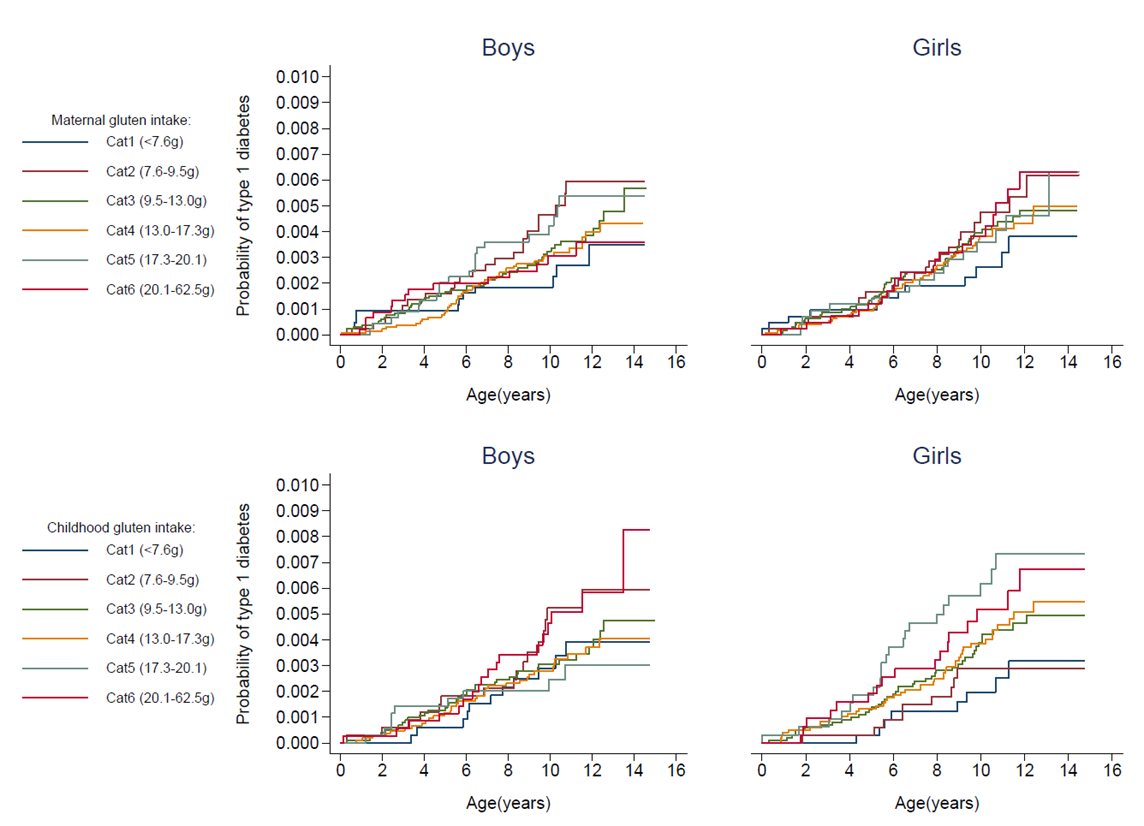
**


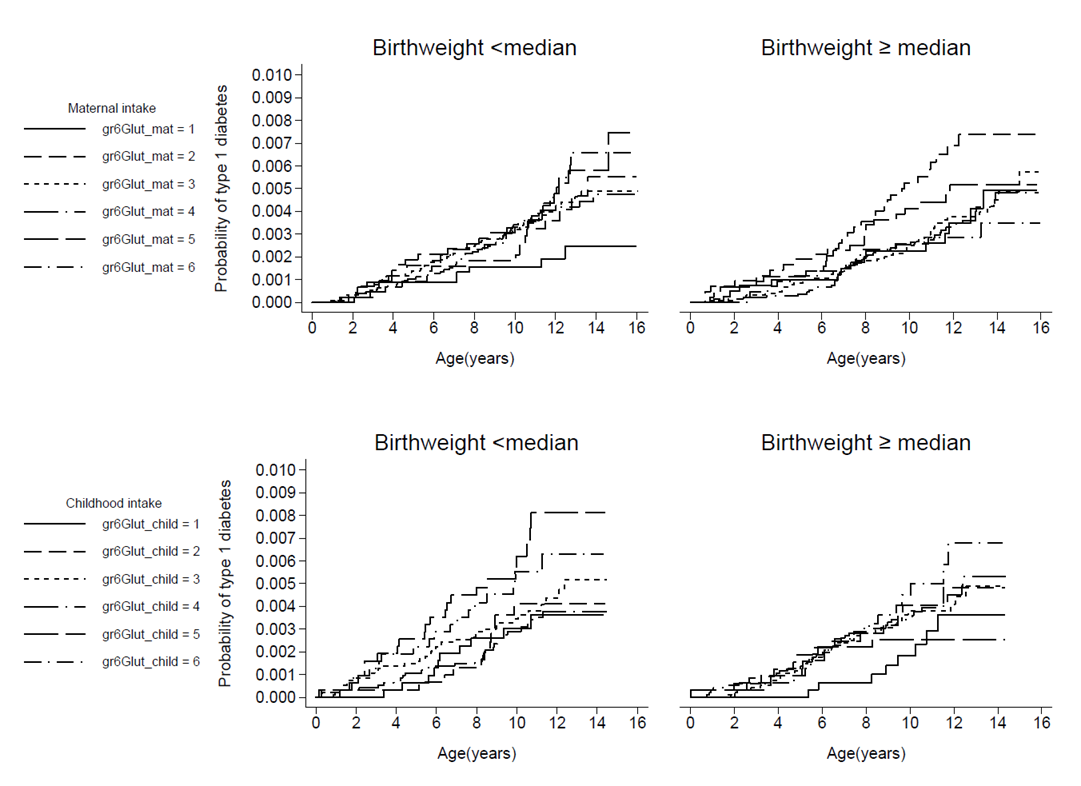

Supplement: S5 Fig — (DOCX) [file pmed.1003032.s012.docx]
