# Supplementary material for: Maternal and child gluten intake and association with type 1 diabetes: The Norwegian Mother and Child Cohort Study
Source: PLoS Med. 2020 Mar 2;17(3):e1003032. doi: 10.1371/journal.pmed.1003032 (PMC7051049; doi:10.1371/journal.pmed.1003032)
Supplement: S1 Text — (DOCX) [file pmed.1003032.s013.docx]

**S1 Text. Analysis plan**

**Analysis plan for project on gluten intake and risk of type 1 diabetes**

By Nicolai A. Lund-Blix, 02.10.18

(with contributions by German Tapia, Ketil Størdal and Lars C. Stene)

Working title:

**Maternal and child gluten intake and risk of type 1 diabetes in children from a Norwegian national prospective cohort study**

**Background**

A paper in the BMJ from the Danish National Birth Cohort (DNBC) study found that a high maternal amount of gluten intake during pregnancy was associated with increased risk of type 1 diabetes (T1D) in the offspring (1). This was the first report of such an association, which supported previous work in the experimental model NOD mice (2). In an accompanying editorial (3), Miettinen & Virtanen raised the possibility that mothers with high gluten intake might provide a high gluten diet to their children, suggesting that part of the observed association may be mediated by child’s gluten intake. Amount of gluten in the child’s diet has long been hypothesized to influence the risk of type 1 diabetes (4), but prospective large scale study have been published. Given the similar design and sample size of DNBC and the Norwegian Mother and Child Cohort study (MoBa) (5), we seek to replicate the finding from the Danish study. In contrast to DNBC, MoBa also collected data on dietary intake in the children, allowing us to also investigate child’s gluten amount in the diet in relation to later risk of T1D. Further, in response to comments (online comments to the BMJ) on the Danish study, we will investigate the potential role of dietary fibre, which tends to be associated with amount of gluten in the diet. While this these research questions has been planned in the MoBa/PAGE study, we tailor our statistical analysis to replicate that of the Danish study to make results directly comparable. A few additional improvements will be done as indicated below. If in doubt, we will follow principles of analysis that we have established in our previous analyses of environmental factors in relation to risk of T1D or celiac disease in the MoBa study as part of the PAGE substudy in MoBa, see e.g. Tapia et al. Int J Epidemiol 2018 (6) or Magnus et al. Epidemiology 2018 (7), which includes imputing missing covariates but not imputing the main exposure(s).

**Aim**

This project aims to investigate the association between the maternal gluten intake during pregnancy, child’s gluten intake at age 18 months, and the risk of type 1 diabetes in the child.

**Main research questions:**

1. Whether a high intake of gluten in maternal diet during pregnancy is associated with a higher risk of T1D in the child.
2. Whether a high intake of gluten in the child’s diet at 18 months of life is associated with a higher risk of T1D in the child.

- Secondary research questions:

1. Whether intake of fiber in maternal diet during pregnancy is associated with a higher risk of T1D in the child.
2. Whether intake of gluten from refined grains in maternal diet during pregnancy is associated with a higher risk of T1D in the child.

**Material and methods**

Study sample:

114.000 mother and child pairs from the Norwegian mother and child cohort study.

Exclusion criteria will be incomplete exposure information, children with an unlikely high gluten intake, other types of diabetes, and maternal type 1 diabetes or celiac disease.

Main exposure:

Total intake of gluten reported by food frequency questionnaires at 22 weeks of pregnancy and at 18 months of age. Use the MoBa food database, official tables and tools, recipes and product labels to estimate the protein content from wheat, barley and rye. Use a conversion factor of 0.75 for gluten content.

As specified in the statistical analysis section, we will use a test for trend with the main exposure as a continuous variable as the primary test, while also performing categorical analysis according to the grouping done in the Danish study (1) to explore potential deviation from (log-) linearity.

Outcome:

Use the Norwegian Childhood Diabetes Registry to obtain date of diagnosis of T1D in the child. The main outcome is time to clinical diagnosis of type 1 diabetes in the child.

Covariates:

To be able to compare our study with the newly published Danish study it is important to use similar covariates in one model: maternal BMI, age, parity, maternal smoking, education level, duration of breastfeeding, caesarean section, energy intake and child’s gender.

In our main model, we will be able to adjust for maternal and child’s gluten intake (depending on the exposure) and add other relevant covariates as: child’s age at gluten introduction, birthweight, prematurity, weight gain first year of life and maternal fibre intake during pregnancy. In the Danish study they do not have data on childhood intake of gluten, and this could be correlated. Maternal intake could be a confounder in the analyses of childhood intake and the childhood intake could be a mediator in the analyses of maternal intake. The relation between maternal gluten intake during pregnancy and childhood intake needs to be explored in this study since we have data on both.

Unlike the Danish study we also have data childhood coeliac disease, but based on the prior work with CD data this variable will reduce the number of cases in the main analyses, and should be included in a secondary model.

Statistical analysis:

We will use Stata v.15 for the statistical analyses, documenting the whole pipeline in do-files (scripts). Two or more independent investigators will check the scripts for accuracy before submission of results (German Tapia, Nicolai A. Lund-Blix, Ketil Størdal and Lars C. Stene will contribute to the analyses). We will use Cox regression analysis to estimate hazard ratios with 95% confidence intervals. Statistical significance is defined as p-values 0.05 or 95% confidence intervals for the hazard ratio not including 1.00. The first analysis will be a test for linear trend based on a dataset where we use multiple imputation with chained equations. We should use per 10 grams increase in gluten intake per day like the newly published Danish study for comparison. To test non-linearity we will analyze gluten intake as a categorical variable. Here we should use the same percentiles as the Danish study for comparison.

The proportional hazards assumption will be assessed using log-minus log plots and testing Schoenfeld residuals. We need to use robust cluster variance estimation to account for potential correlation among siblings.

Statistical power

Using results from the Danish study we used the program Epitome Power v3.0 (8) to estimate the power for a scenario with exposure distribution as in the Danish study and a similar strength of relation between exposures and T1D. (Power is based on logistic regression model, which gives very similar results as the Cox model for rare disease such as T1D). The estimated power is 94% and 88% for a test for trend if there is a true relative risk of 2.00 comparing the lowest intake percentile with the highest intake percentile of maternal and childhood intake, respectively. Due to the number of cases and experiences from the past works with this data, stratification into subgroups and use of small categories should be avoided even if our number is high compared to other studies in the field.

Suggested sensitivity analysis:

1. Complete case analysis of the main analyses (Since we are using imputed data in the main statistical analyses).
2. Analyses of maternal fiber intake and gluten intake from refined grains during pregnancy and risk of type 1 diabetes in the child.
3. Further adjust main analyses for child coeliac disease.

**References**

1. Antvorskov JC, Halldorsson TI, Josefsen K, Svensson J, Granstrom C, Roep BO, et al. Association between maternal gluten intake and type 1 diabetes in offspring: national prospective cohort study in Denmark. BMJ. 2018;362:k3547.

2. Hansen CH, Krych L, Buschard K, Metzdorff SB, Nellemann C, Hansen LH, et al. A maternal gluten-free diet reduces inflammation and diabetes incidence in the offspring of NOD mice. Diabetes. 2014;63(8):2821-32.

3. Miettinen ME, Virtanen SM. Dietary gluten and type 1 diabetes. BMJ. 2018;362:k3867.

4. Antvorskov JC, Josefsen K, Engkilde K, Funda DP, Buschard K. Dietary gluten and the development of type 1 diabetes. Diabetologia. 2014;57(9):1770-80.

5. Olsen SF, Birgisdottir BE, Halldorsson TI, Brantsaeter AL, Haugen M, Torjusen H, et al. Possibilities and considerations when merging dietary data from the world's two largest pregnancy cohorts: the Danish National Birth Cohort and the Norwegian Mother and Child Cohort Study. Acta Obstet Gynecol Scand. 2014;93(11):1131-40.

6. Tapia G, Størdal K, Mårild K, Kahrs CR, Skrivarhaug T, Njølstad PR, et al. Antibiotics, acetaminophen and infections during prenatal and early life in relation to type 1 diabetes. Int J Epidemiol. 2018;47(5):1538-48.

7. Magnus MC, Tapia G, Olsen SF, Granstrom C, Mårild K, Ueland PM, et al. Parental smoking and risk of childhood-onset type 1 diabetes. Epidemiology. 2018;29(6):848-56.

8. García-Closas M, Lubin JH. Power and sample size calculations in case-control studies of gene-environment interactions: comments on different approaches. Am J Epidemiol. 1999;149:689-92.
